# Incorporation of Comonomer *exo*-5-(Diphenylphosphato)Isosorbide-2-*endo*-Acrylate to Generate Flame Retardant Poly(Styrene)

**DOI:** 10.3390/polym11122038

**Published:** 2019-12-09

**Authors:** Bob A. Howell, Yoseph G. Daniel

**Affiliations:** Science of Advanced Materials Center for Applications in Polymer Science, Department of Chemistry and Biochemistry, Central Michigan University, Mt. Pleasant, MI 48859-0001, USA; danie1yg@cmich.edu

**Keywords:** flame retardant poly(styrene), phosphatoisosorbide acrylate, reactive phosphorus flame retardants, phosphorus-containing vinyl monomers, flame retardant vinyl monomers, flame-retarding copolymerization, biobased flame retardants

## Abstract

A phosphorus containing acrylate monomer has been constructed from isosorbide, a renewable biomaterial. Treatment of isosorbide with diphenylchlorophosphate generates a mixture of phosphorus esters from which *exo*-5-(diphenylphosphato)isosorbide-2-*endo*-ol may be isolated using column chromatography. Conversion of the alcohol to the corresponding acrylate by treatment with acroyl chloride provides a reactive acryloyl monomer containing a diphenylphosphato unit. Copolymerization of this monomer, at levels to provide 1% or 2% phosphorus incorporation, with styrene generates a polymer with substantially diminished flammability compared to that for styrene homopolymer.

## 1. Introduction

In the decades since World War II, the development of polymeric materials has been a major contributor to the well-being of modern society [1,2]. These materials are pervasive in everyday life from food packaging to medicines to home construction to transportation. For most applications, these materials must be flame retarded [3]. Most commonly, flame retardant additives are introduced during polymer processing [4,5]. Less often, reactive flame retardant compounds are covalently incorporated into the polymer during polymerization. This is a particularly attractive approach for suitably-modified vinyl monomers [6,7,8,9,10,11]. Many flame retardants function by promoting the formation of protective char layer at the surface of the degrading polymer, while others release volatile components to the gas phase which scavenge combustion-propagating radicals [12]. Some may also intercept radicals leading to particulate formation and act as smoke suppressants [13]. Traditional organohalogen flame retardants are of the latter type. Among these, brominated aromatics have been most commonly used. These materials are readily available, modest in cost and effective as flame retardants [14,15]. They function by releasing volatile hydrogen bromide, formed by interaction with the thermally-degrading polymer, to the gas phase where it traps radicals which propagate the combustion process [12]. The action of these compounds is enhanced by the presence of antimony oxide. Volatile antimony bromide and oxybromides are formed which carry bromine to the gas phase [12,15]. Because of the toxicity of antimony oxide, alternative agents are being sought to serve in this role [16]. Although organohalogen compounds have long been widely used as flame retardants, this use is coming under increasing societal and regulatory pressure [17,18]. These compounds tend to migrate from polymeric matrices into which they have been incorporated and enter the environment [19,20]. They are stable, persistent in the environment, and bioaccumulate. Because of the widespread distribution, human exposure is relatively constant. This exposure has been associated with the development of a number of disease states [21,22]. Because of the negative health impacts of organohalogen flame retardants, organophosphorus compounds are increasingly the focus of new developments [3,23,24,25,26]. Organophosphorus flame retardants may function in either the solid phase or the gas phase depending largely on the level of oxygenation at phosphorus [27,28,29,30]. Compounds containing phosphorus at a high level of oxygenation at phosphorus, e.g., phosphates, particularly alkyl phosphates, eliminate a phosphorus acid when undergoing decomposition in a degrading polymer matrix. The phosphorus acid promotes cationic crosslinking and char formation at the surface of the degrading polymer [27,28,29,30]. This char layer acts as an insulation barrier to reduce heat feedback from the flame and consequent pyrolytic formation of volatile fuel fragments to feed the flame. Combustion is diminished for lack of fuel. In contrast, compounds containing phosphorus at low levels of oxygenation, e.g., phosphinates or phosphonates, tend to decompose to extrude PO radical to the gas phase where they scavenge combustion propagating radicals [31,32]. Some organophosphorus compounds may display both condensed phase and gas phase activity, particularly those containing two types of phosphorus functionality [33,34,35]. In general, organophosphorus compounds are less toxic than organohalogen counterparts [36,37,38,39]. Toxicity of organophosphorus compounds is usually the consequence of a particular structure rather than a function of the compounds as a class. Organophosphorus compounds based on renewable biosources are particularly attractive [40,41,42,43,44,45,46,47,48,49]. The precursors to these compounds are usually nontoxic, benign, or biodegradable in the environment, often renewable annually, and available at costs that are independent of fluctuations in petrochemical markets. These precursor materials may come from a variety of sources from food waste to seed grains [49]. The ultimate source for many of these compounds is starch derived from cereal grains. In the US, corn alone may provide a base for a wide variety of useful compounds [50,51]. One of the compounds available from starch is isosorbide. Hydrolysis of starch affords glucose which may be reduced and dehydrated to produce isosorbide, a diether diol [52]. Its difunctionality makes it an attractive biobase for the generation of both polymers and polymer additives [53]. The two hydroxyl groups display different reactivity. One is exo and the other endo to the interior of the molecule. In this case, isosorbide was converted into a mixture of three diphenylphosphate esters, two monoesters, and the diester, which could be separated by column chromatography. *exo*-5-(Diphenylphosphato)isosorbide-2-*endo*-ol was converted to the corresponding acrylate to provide a vinyl monomer containing phosphorus. Copolymerization of this monomer with styrene at levels to provide 1% and 2% of phosphorus incorporation provided polymers with dramatically reduced flammability with respect to that for the styrene homopolymer.

## 2. Experimental

### 2.1. Methods and Instrumentation

In general ester synthesis and polymerization were carried out using the previously described methods [44,45]. Characterization was accomplished using chromatographic, thermal, and spectroscopic methods as previously described [44,45,53,54]. Thermal transitions were determined using differential scanning calorimetry (DSC) at a heating rate of 10 °C/min and a TA Instruments Q2000 instrument (TA Instruments, New Castle, DE, USA). The DSC cell was purged with dry nitrogen at 50 mL/min during analysis. Thermal decomposition behavior was evaluated using thermogravimetry (TGA) (TA Instruments, New Castle, DE, USA). A TA Instruments Q500 unit and samples of 6–10 mg contained in a platinum pan were used. The heating rate was 10 °C/min. The sample compartment was purged with dry nitrogen at 50 mL/min during analysis.

### 2.2. Materials

Common solvents and reagents were obtained from ThermoFisher Scientific (Waltham, MA, USA) or the Aldrich Chemical Company (Milwaukee, WI, USA). Tetrahydrofuran was distilled from lithium aluminum hydride in a nitrogen atmosphere prior to use; methylene chloride from calcium hydride. Isosorbide, acryloyl chloride, triethylamine, and azobisisobutyronitrile (AIBN) were from the Aldrich Chemical Company. Diphenylchlorophosphate was supplied by ICL-IP America, Inc. (Ardsley, NY, USA).

### 2.3. Synthesis

**Scheme 1:**
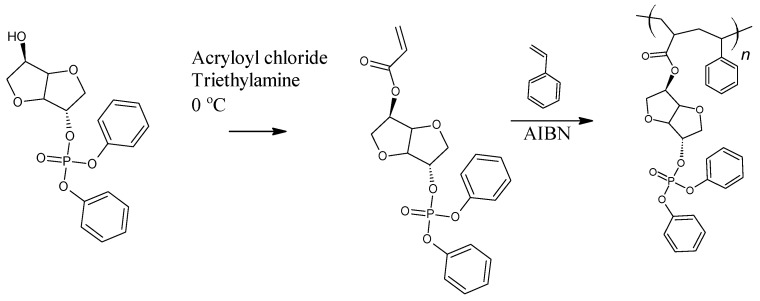
Preparation of *exo*-5-(Diphenylophosphato)isosorbide-2-*endo*-acrylate/Styrene Copolymers.

Isosorbide esters were prepared as previously described [44,45,53]. The polymerization is outlined in Scheme 1 and described below.

#### 2.3.1. *exo*-5-(Diphenylphosphato)isosorbide-2-*endo*-ol

Treatment of a solution of isosorbide and triethylamine with diphenylchlorophosphate generated a mixture of esters (two monoesters and the diester). *exo*-5-(Diphenylphophato)isosorbide-2-*endo*-ol was isolated as a colorless oil using column chromatography (silica gel; 90:10 hexane/ethyl acetate as eluent).

#### 2.3.2. *exo*-5-(Diphenylphosphato)isosorbide-2-*endo*-Acrylate

To a solution of 8.44 g (22.3 mol) of *exo*-5diphenylphosphato)isosorbide-2-*endo*-ol and triethylamine (4.35 mL, 3.16 g) in 80 mL of anhydrous THF stirred near 0 °C (external ice bath) in a nitrogen atmosphere was added, dropwise, over a period of 0.5 h, 2.35 mL (26.8 mmol) of acryloyl chloride. After the additive was complete, the mixture was allowed to warm to room temperature and stirred until the reaction was complete. Progress of the reaction was monitored by periodic removal of an aliquot of the mixture for analysis using infrared spectroscopy. The reaction was deemed to be complete when the spectrum of the mixture no longer contained hydroxyl absorption. Triethylammonium chloride was removed by filtration and the volume of solvent was reduced by rotary evaporation at reduced pressure. The concentrated solution was diluted with 100 mL of methylene chloride and washed, successively, with two 40 mL portions of 10% aqueous sodium hydroxide solution, 40 mL of 10% aqueous hydrochloric acid solution and 40 mL of saturated aqueous sodium chloride solution. The solution was dried over anhydrous sodium sulfate and the solvent was removed by rotary evaporation at a reduced pressure to afford the acrylate as a colorless oil (9.51 g, 93.2% yield).

### 2.4. Polymerization

In general, copolymers of diphenylphosphateisosorbide acrylate and styrene were prepared by dissolving the appropriate amount of monomers (to provide copolymers containing 1 or 2% wt % phosphorus) and initiator (azobisisobutyronitrile, AIBN, 1 mol%) in chloroform. The solution was degassed using dry nitrogen and stirred under nitrogen at 65 °C until all unsaturation had been consumed as evidenced by lack of the corresponding absorbance in the infrared spectrum of an aliquot of the reaction mixture removed for inspection. The solution was poured into rapidly stirred hexane (10-fold excess) to precipitate the polymer. The polymer was collected by filtration at reduced pressure and dried at 50 °C and 15 torr. Copolymer dispersity and molecular weight were determined using size exclusion chromatography. Copolymer composition was determined using either proton or quantitative carbon-13 NMR spectroscopy. In a typical example, (for 2 wt % phosphorus in the polymer) 2.30 g (4.5 mmol) of *exo*-5-(diphenylphosphato) isosorbide-2-*endo*-ol, 12.0 g (115.2 mmol) of styrene and 0.50 g of AIBN in 70 mL of chloroform was stirred at 65 °C for 12 h. The solution was poured into 600 mL of rapidly stirred hexane. The polymer was collected and dried as noted above: *T*_g_ 85 °C (DSC); *M*_n_ 13, 600, *M*_w_ 26, 600, *M*_w_/*M*_n_ 1.9 (size exclusion chromatography (SEC); calibration with narrow molecular weight poly(styrene) standards); 7.15 mol % acrylate from proton NMR-integration of the peak area for phenyl protons versus that for the acrylate methylene protons or from integration of the corresponding peaks in the quantitative 13C NMR spectrum.

### 2.5. Flammability Assessment

Microscale combustion calorimetry (MCC) was carried out using a Fire Testing Technology, Ltd. (East Grinstead, UK) instrument according to a standard protocol (ASTM D7309-072a).

## 3. Results and Discussion

**Figure 1:**
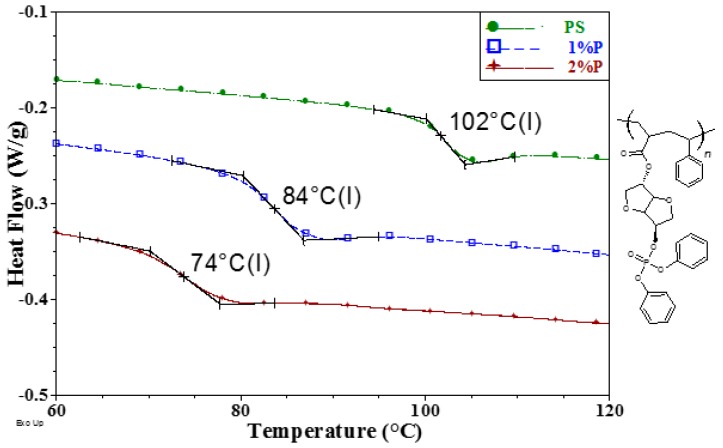
Glass Transition Temperatures for *exo*-5-(Diphenylphosphato)isosorbide-2-*endo*-acrylate/Styrene Copolymers.

**Figure 2:**
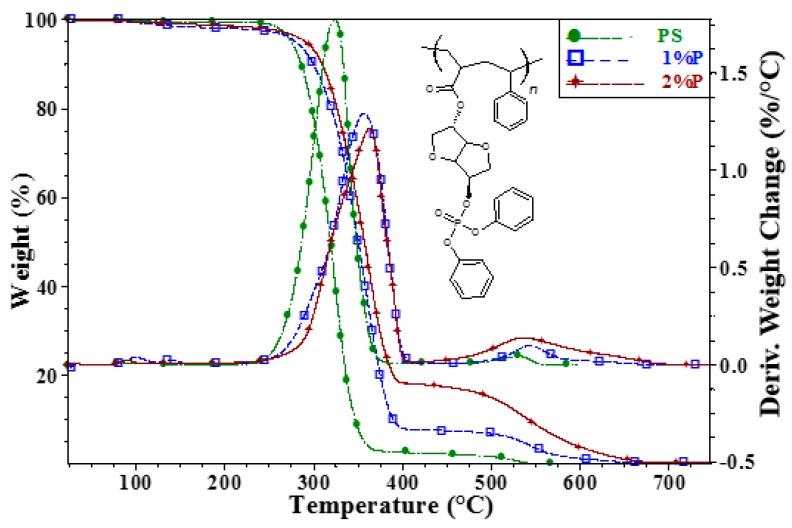
Thermal Stability of *exo*-5-(Diphenylphosphato)isosorbide-2-*endo*-acrylate/Styrene Copolymers.

The development of polymer additives from renewable bioresources continues to grow at an increasing rate (now about 20%/year) [55]. This is largely driven by concern about the release of toxic agents to the environment and the likely negative impact of these materials on human health. Flame retardants, in particular have been a focus of much of this development [40,41,42,43,44,45,46,47,48,49]. Precursors for biobased flame retardants may come from a variety of sources including plant oils [49], seed grain constituents [50,51], and by-products of food manufacture [47,49]. Startch from a variety of crop grains is an extremely valuable source of several useful compounds. [50,51] It may be readily hydrolyzed to generate glucose which may be converted to hydroxymethylfurfural, a base for the production of a large number of compounds. Alternatively, glucose may be reduced and subjected to double dehydration to form isosorbide, a diether diol. Isosorbide has been used as a platform for the generation of a variety of effective flame retardants [44,45,46,56]. Isosorbide has now been converted to the mono(diphenylphosphato)ester from which an acrylate monomer can be generated. Copolymerization of the acrylate (at levels to incorporate 1 and 2 wt % phosphorus into the polymer) with styrene afford polymers with much diminished flammability compared to that of unmodified poly(styrene). The process is illustrated in Scheme 1. Copolymer composition determined using either proton or quantitative 13C NMR spectroscopy indicated that the expected level of acrylate, and therefore, the proper level of phosphorus was incorporated in all cases. Molecular weights (*M*_n_) as determined using SEC were 12–15,000 with a dispersity of about 2.0. As expected for a polymer containing large pendant groups, the glass transition temperatures (DSC) for the copolymers are somewhat lower than that for the homopolymer (Figure 1). It may be expected that the incorporation of comonomer at the levels indicated will not significantly impact the properties of poly(styrene). As determined using thermogravimetry, the thermal stability of the copolymers (Figure 2) is slightly greater than that for poly(styrene). The onset for decomposition of poly(styrene) is 250 °C. With the introduction of comonomer, both of these values increase to 275 and 350 °C for the copolymer containing 1% phosphorus, and to 300 and 375 °C for copolymer containing 2% phosphorus. While these increases in degradation temperature are small, they may reflect radical scavenging in the degrading polymer [57]. The residual char was 1% of the initial sample mass for poly(styrene). This increased to 7% for copolymer containing 1% phosphorus, and to 17% for that containing 2% of phosphorus. This is as would be expected for the presence of an entity containing phosphorus with a high level of oxygenation and suggests that flame retardancy arises from condensed phase activity [27,28,29,30].

**Figure 3:**
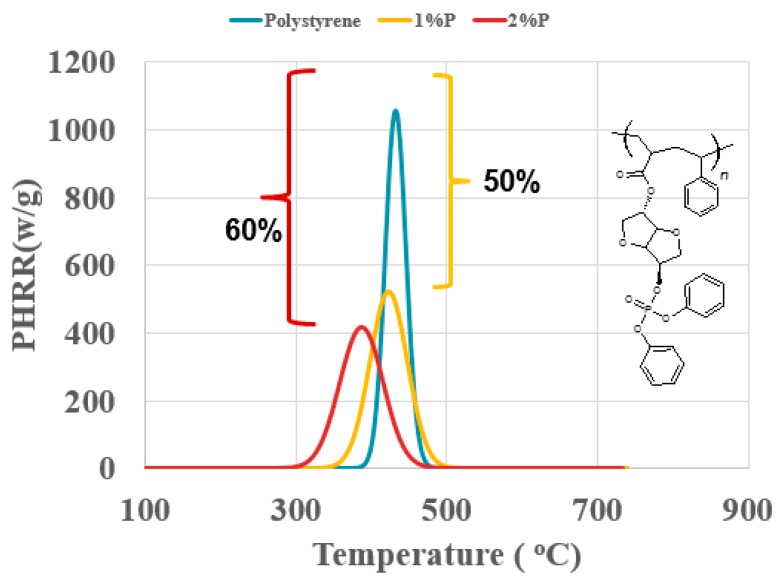
Heat release rate for combustion of *exo*-5-(Diphenylphosphato)isosorbide-2-*endo*-acrylate/Styrene Copolymers.

To assess the flammability of the copolymers, microscale combustion calorimetry (MCC) was used. MCC has been shown to be an effective and convenient method for assessing flammability [58,59,60]. As may be noted from Figure 3, the presence of 1% phosphorus in the polymer has a dramatic impact on peak heat release rate, a 50% reduction compared to that for homopolymer poly(styrene). This is a huge impact and illustrates the effectiveness of incorporation of a biobased phosphorus-containing comonomer into a standard vinyl polymer.

Poly(styrene) containing no flame-retarding entity is highly flammable. The dramatic reduction in the peak heat release rate upon incorporation of a low-level of phosphorus-containing comonomer certainly illustrates the effectiveness of this approach for reducing flammability. Since acrylate is broadly reactive toward vinyl monomers, this approach could be useful for flame-retarding a variety of polymers.

## 4. Conclusions

Isosorbide is a diether diol obtained from glucose (starch). It may be converted, first, to a diphenylphosphate at one hydroxyl group and then to an acrylate at the other to provide a bioderived vinyl monomer. Incorporation of low levels of this acrylate as a comonomer into poly(styrene) produces a material with strongly reduced flammability compared to that for the homopolymer. The presence of sufficient comonomer to introduce 1 wt % phosphorus brings about a 50% reduction in peak heat release rate for combustion of the polymer. This approach to the introduction of flame retardancy should be broadly applicable to vinyl polymers.
